# Topical application of *Zanthoxylum piperitum* extract improves lateral canthal rhytides by inhibiting muscle contractions

**DOI:** 10.1038/s41598-020-78610-w

**Published:** 2020-12-09

**Authors:** Wooseon Hwang, Daehyun Kim, Oh Sun Kwon, Yun-Sun Kim, Byungjun Ahn, Nae-Gyu Kang

**Affiliations:** R&D Center, LG Household and Healthcare, E10 building, LG Science Park, 70 Magokjungang-10-ro, Seoul, 07911 South Korea

**Keywords:** Biological techniques, Neuroscience, Physiology

## Abstract

Facial wrinkles are the predominant phenotypes of skin aging. To date, one of the most effective ways to improve wrinkles is botulinum toxin type A (BoNT/A) injection, which inhibits muscle contractions by reducing acetylcholine release from neurons. However, since BoNT/A is a hazardous neurotoxin, the injection can only be performed by medical doctors and the procedure is only possible through invasive injection, causing inconveniences such as pain. To overcome these inconveniences, we tried to find a way to reduce wrinkles non-invasively via mechanisms similar to BoNT/A. We first designed in vitro assays to test BoNT/A-like muscle contraction inhibition in two different model systems. By using the assays, we identified *Zanthoxylum piperitum* (*Z. piperitum*) fruit extract as a BoNT-like reagent (27.7% decrease of muscle contraction rates by 1000 ppm of *Z. piperitum* extract treatment). Next, we determined mechanisms of how *Z. piperitum* extract decreases muscle contraction rates and found that the extract treatment inhibits electrical signal transduction in neurons. We also showed that among known components of *Z. piperitum* extract, quercitrin is responsible for muscle contraction inhibition. We further identified that *Z. piperitum* extract has synergistic effects with acetyl hexapeptide-8 and BoNT/A light chain, which are well-known BoNT-like peptides. Finally, we showed that topical treatment of the *Z. piperitum* extract indeed decreases facial wrinkles and treatment of *Z. piperitum* extract with acetyl hexapeptide-8 has a tendency to improve wrinkles synergistically (14.5% improvement on average). The synergistic effect of the combination is expected to improve wrinkles effectively by implementing the BoNT/A mechanisms in a non-invasive way.

## Introduction

Facial wrinkles are the predominant and visible phenotypes of skin aging. Wrinkles are classified by 4 types according to their anatomical characteristics^1^. The 4 types of wrinkles are atrophic, elastotic, expressional, and gravitational wrinkles. Among them, expressional wrinkles have distinct patterns according to the forces imposed by facial expression muscles^1^. Lateral canthal rhytides (Crow’s feet) are the representative example of the expressional wrinkles.

Botulinum toxin type A (BoNT/A) injection is one of the most common and effective procedures to reduce facial wrinkles, especially for expressional wrinkles^2^. BoNT/A improves wrinkles by inhibiting muscle contractions. BoNT/A reduces acetylcholine release from neuronal cells through cleaving SNARE (SNAp REceptor) complex component SNAP-25 (Synaptosomal-Associated Protein with 25 kDa size). The reduction of the acetylcholine release causes muscle contraction inhibition and prevents exaggeration of the experimental wrinkles^2^.

Although the BoNT/A injection is effective to treat wrinkles, the procedure can only be performed by medical doctors since BoNT/A is a neurotoxin. Moreover, the injection procedure is only possible through invasive injection. To overcome these inconveniences, dermatologists and the cosmetics researchers have made efforts to improve wrinkles via mechanisms similar to that of BoNT/A in a non-invasive way. For example, Lipotec, Ltd. developed an acetyl hexapeptide-8 as a BoNT-like wrinkle-reducing ingredient^3^. The acetyl hexapeptide-8 is composed of a short sequence of SNAP-25 so that it competitively binds to the SNARE complex instead of SNAP-25 to inhibit SNARE formation. Another BoNT-like cosmetic ingredient is a light chain of Botulinum toxin (BoNT/A-LC) tagged with transdermal peptide TD1 (Transdermal Peptide-1)^4^ or TAT (TransActivator of Transcription peptide)^5^. Despite these efforts, the identification of novel muscle contraction-inhibitory chemicals is still challenging. For example, since non-mammal or in vitro methods to test muscle contraction inhibition are scarce, it is hard to test candidate materials.

## Results

**Figure 1:**
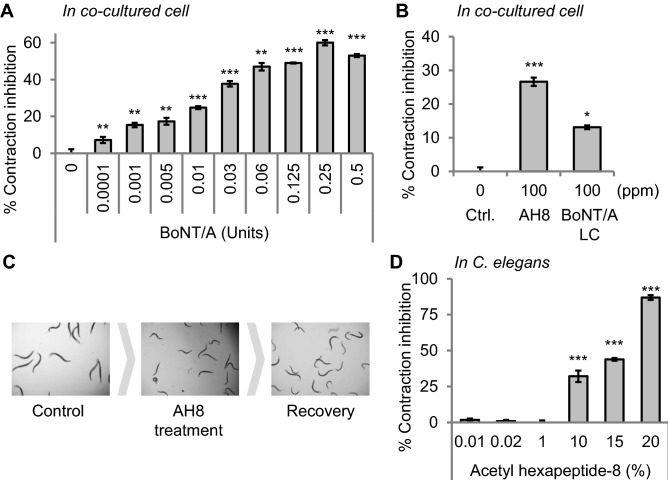
Muscle contraction assays in the co-cultured cell system and *C. elegans* system. (**A**) Validation of the muscle contraction assay in a co-cultured cell system. Botulinum toxin type A (BoNT/A) treatment inhibited muscle contraction in a dose-dependent manner. (**B**) Acetyl hexapeptide-8 (AH8) and BoNT/A light chain (BoNT/A LC), which are known as BoNT-like reagents also inhibited muscle contraction in cells. (**C**) Muscle contraction inhibition by acetyl hexapeptide-8 (AH8) treatment was reversible in *C. elegans*. Ten percent of acetyl hexapeptide-8 fully inhibited the movement of *C. elegans*, and the paralysis was completely recovered after removing chemicals by simple washing. (**D**) Acetyl hexapeptide-8 treatment inhibited muscle contraction in *C. elegans* as well. For the muscle contraction assays*,* all the experiments were performed at least twice. Error bars represent standard error of mean (S.E.M., *p < 0.05, **p < 0.01, ***p < 0.001, two‐tailed Student's t‐test).

To identify compounds that inhibit muscle contraction, we first designed muscle contraction assays in two different model systems, a co-cultured cell system, and a *Caenorhabditis elegans* (*C. elegans*) system. First, we validated the cell culture system-based assay with the Botulinum toxin type A (BoNT/A) treatment and found that BoNT/A treatment inhibits contraction of muscle cells in a dose-dependent manner (Fig. 1A). We also tested known BoNT-like reagents, the acetyl hexapeptide-8 and light chain part of the BoNT/A (BoNT/A LC), and found that 100 ppm of acetyl hexapeptide-8 and BoNT/A LC inhibits muscle contractions 26% and 13%, respectively (Fig. 1B and Supplementary Video 1 and 2). These data also validated the assays.

To examine the muscle contraction at the organismal level, we used *C. elegans* as a model. *C. elegans* is widely used as an experimental model especially in the neuroscience field because the nervous system is much simpler than mammals^6,7^. In addition, a previous study showed that BoNT/A treatment could paralyze *C. elegans*^8^*,* indicating that the pathways required for the BoNT/A-mediated muscle contraction inhibition are conserved in *C. elegans*. Also, changes in muscle activity or contraction can be easily monitored by examining body movements. We treated worms with acetyl hexapeptide-8 and found that acetyl hexapeptide-8 treatment decreases the movement of worms does-dependently (Fig. 1C, D, and Supplementary Video 3 and 4). With the data, we also validated the muscle contraction assays in the *C. elegans* system.

**Figure 2:**
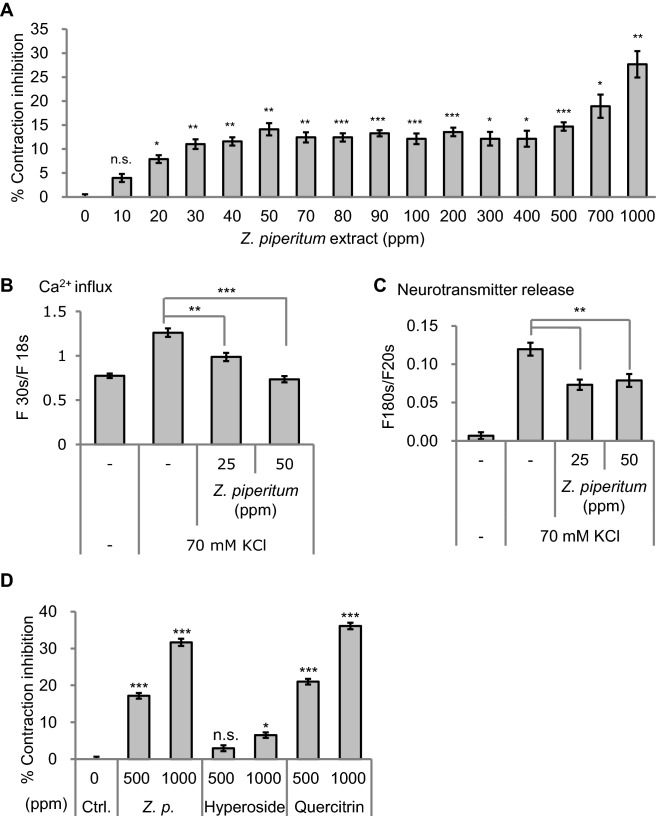
Identification of *Z. piperitum* extract as a BoNT-like ingredient. (**A**) *Zanthoxylum piperitum* (*Z. piperitum*) extract treatment inhibited muscle contraction rates in a dose-dependent manner. (**B** and **C**) Mechanisms of how *Z. piperitum* extract inhibits muscle contraction. *Z. piperitum* extract treatment reduced Ca^2^^+^ ion influx into the neuronal cells (n = 5) (**B**), resulting in a decrease in neurotransmitter release (n = 7) (**C**). (**D**) Effects of hyperoside or quercitrin treatment on muscle contraction rates. Hyperoside treatment had little or no effect on muscle contraction, however, quercitrin treatment inhibited muscle contraction. All the muscle-contraction assays were repeated at least twice. *Z. p.* indicates *Z. piperitum* extract. Error bars represent standard error of mean (S.E.M., *p < 0.05, **p < 0.01, ***p < 0.001, two‐tailed Student's t‐test).

Next, we tested candidate chemicals for muscle contraction inhibition. Among candidates, we focused on the *Zanthoxylum piperitum (Z. pipertium)*. *Z. piperitum* has been traditionally used as a pain-relieving herb in Asian countries including Korea and the anti-nociceptive activity is experimentally confirmed^9^. Besides, *Z. piperitum* tastes tingling and numbing, similar to the symptoms of weak paralysis. Considering these characteristics*,* we hypothesized that *Z. piperitum* extract treatment could inhibit muscle contraction. Indeed, the treatment of *Z. piperitum* extract inhibited muscle contraction in *C. elegans* dose-dependently (Fig. 2A). One thousand parts-per-million (ppm) of *Z. piperitum* extract reduced 30% of muscle contraction rates, which is similar to the effect of 10% of acetyl hexapeptide-8 treatment. This result suggests that *Z. piperitum* extract can act as a BoNT-like compound.

To determine how *Z. piperitum* extract treatment inhibits muscle contraction rate, we tested whether *Z. piperitum* extract regulates signal transduction in neurons. We found that *Z. piperitum* extract treatment reduced Ca^2+^ influx in neuronal cells (Fig. 2B), and as a result, reduced neurotransmitter release (Fig. 2C). These data indicate that *Z. piperitum* extract inhibits muscle contraction by attenuating electric signal transduction in presynaptic neurons.

Next, we examined which components in the *Z. piperitum* extract are responsible for muscle contraction inhibition. Among the previously reported components of *Z. piperitum* extract^10^, we tested the muscle contraction-inhibitory effects of two major compounds, quercitrin and hyperoside. We found that quercitrin treatment decreased the muscle contraction rates but hyperoside treatment had little or no effect (Fig. 2D), suggesting that quercitrin is responsible for muscle contraction inhibition.

To enhance the wrinkle-reducing effects of BoNT-like compounds, we tried to maximize the muscle contraction-inhibitory effects of *Z. piperitum* extracts. Based on the aforementioned findings that *Z. piperitum* extract treatment reduced muscle contraction rate by inhibiting Ca^2+^ influx into neurons, we designed two strategies to enhance the muscle contraction-inhibitory effects of *Z. piperitum* extract. First, other BoNT-like compounds that target different steps at the signal transduction or transmission might have synergistic effects with *Z. piperitum* extract. Second, to increase the efficiency of BoNT-like compounds by enhancing skin absorption.

**Figure 3:**
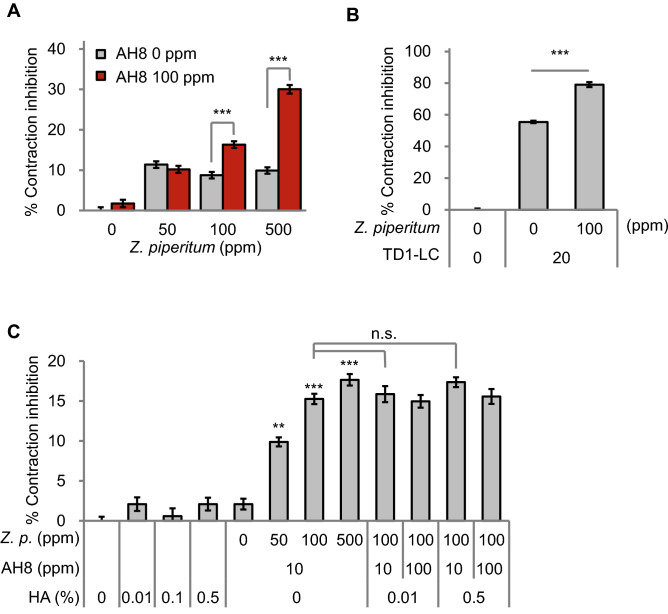
Synergistic effects of *Z. piperitum* extract and BoNT-like ingredients on muscle contraction inhibition. (**A** and **B**) *Z. piperitum* extract treatment synergistically inhibited muscle contraction with acetyl hexapeptide-8 (AH8) (**A**) and TD1-BoNT/A LC (TD1-LC) (**B**). (**C**) Muscle contraction inhibitory effects of the combination of *Z. piperitum* extract (*Z. p.*), acetyl hexapeptide-8 (AH8), and hyaluronic acids (HA). All the assays were performed at least twice. Error bars represent standard error of mean (S.E.M., n.s. not-significant, *p < 0.05, **p < 0.01, ***p < 0.001, two‐tailed Student's t‐test).

For the first approach, we tested two BoNT-like reagents, acetyl hexapeptide-8 and BoNT/A light-chain tagged with TD1 transdermal peptide (TD1-LC), which inhibit SNARE complex formation. Indeed, we found that *Z. piperitum* extract treatment synergistically reduces the muscle contraction rate with acetyl hexapeptide-8 and TD1-LC (Fig. 3A and B). One hundred parts-per-million (ppm) of acetyl hexapeptide-8 treatment had no effect on muscle contraction rate in *C. elegans* (Fig. 3A). On the other hand, when we treat worms with 100 ppm of acetyl hexapeptide-8 together with 100 or 500 ppm of *Z. piperitum* extract, muscle contraction rate decreased more than the reducing effect of the single treatment of *Z. piperitum* extract. We confirmed the synergistic effects of *Z. piperitum* extract and acetyl hexapeptide-8 on muscle contractions in different concentrations (Figure S1A) as well as in a different system (cell culture system, Figure S1B). These results suggest that *Z. piperitum* extract and other BoNT-like chemicals such as acetyl hexapeptide-8 and BoNT/A LC might synergistically reduce wrinkles as well.

For the second approach, we tested ultra-low molecular weight hyaluronic acid which is previously reported to increase the skin absorption of acetyl hexapeptide-8^11^. Consistent with the previous report, hyaluronic acid increased the absorption rate of acetyl hexapeptide-8 (Figure. S2A-C), but it did not further increase the muscle contraction-inhibitory effect of *Z. piperitum* extract and acetyl hexapeptide-8 (Fig. 3C).

**Figure 4:**
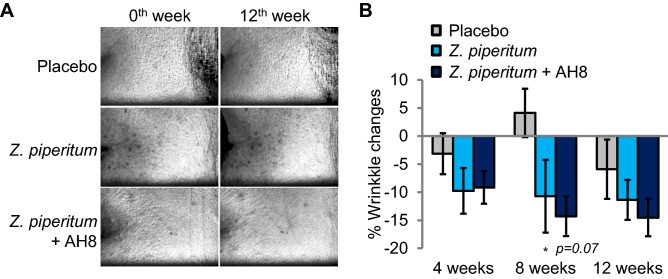
Wrinkle-reducing effect of *Z. piperitum* extract. (**A** and **B**) Improvement of the lateral canthal rhytides (Crow’s feet) by topical treatment of *Z. piperitum* extract and acetyl hexapeptide-8 (AH8) (**A**) and the quantification (**B**). Topical treatment of *Z. piperitum* extract for improved lateral canthal rhytides. In addition, topical treatment of *Z. piperitum* extract and acetyl hexapeptide-8 (AH8) together tended to improve wrinkles synergistically, but the statistics were not significant. Error bars represent standard error of mean (S.E.M., *p < 0.05, two‐tailed Student's t‐test).

Finally, we determined the effect of topical treatment of *Z. piperitum* extract on facial wrinkles (Fig. 4A and B). Compared to placebo treatment, *Z. piperitum* extract treatment for 12 weeks ameliorated lateral canthal rhytides (Crow’s feet) (Fig. 4A). The topical treatment of *Z. piperitum* extract improved 11.4% of lateral canthal rhytides (Fig. 4A and B). We also examined whether *Z. piperitum* extract and acetyl hexapeptide-8 have synergistic effects on wrinkle improvement and found that topical treatment of *Z. piperitum* extract and acetyl hexapeptide-8 together has a tendency to improve wrinkles synergistically (Fig. 4A and B, 14.5% reduction on average). Together, these data indicate that *Z. piperitum* extract treatment can improve wrinkles by inhibiting facial expression muscle contraction, and the combination of *Z. piperitum* extract and acetyl hexapeptide-8 might have synergistic effects on wrinkles.

## Discussion

After we examined the muscle contraction-inhibitory effects and wrinkle-reducing effects of *Z. piperitum* as well as acetyl hexapeptide-8, we questioned whether these cosmetic ingredients can penetrate the skin barrier to reach sensory neurons. Among topically applied acetyl hexapeptide-8, 6.5% of samples were absorbed in the stratum corneum (SC) layer, and total 7.8% of the samples were absorbed in the epidermis and dermis layers including SC (Figure S3). A small portion (0.03%) of the acetyl hexapeptide-8 even penetrates the epidermis and dermis layers (Figure S3). Since nerve endings of the sensory neurons are innervated in the epidermal layers^12–14^, acetyl hexapeptide-8 absorbed in the epidermis and dermis layers is likely to inhibit muscle contraction, and as a result, ameliorates expressional wrinkles.

One of the interesting points is there is a gap between concentrations of acetyl hexapeptide-8 in cell culture systems and in *C. elegans*. For 30% of muscle contraction inhibition, 100 ppm of acetyl hexapeptide-8 is required for cell-based assays. In the case of *C. elegans*, 10% of acetyl hexapeptide-8 is required. This concentration gap might be due to the differences in the excessiveness of chemicals for the neuronal cells. In the case of the co-cultured cell system, since cells are directly exposed to acetyl hexapeptide-8, low concentration (ppm range) might be enough to inhibit muscle contraction. In the case of *C. elegans,* since worms are organisms, acetyl hexapeptide-8 should pass through thick cuticle layers or enter to the cilia of sensory neurons, so much higher concentration might be needed. In humans, 10% of acetyl hexapeptide-8 is required for 30% of wrinkle reduction, similar to the case of *C. elegans*^3^.

Kim et al. previously showed that ultra-low molecular weight hyaluronic acid (0.5–10.1 kDa) promotes skin absorption of acetyl hexapeptide-8^11^. Based on this, we thought that hyaluronic acid may increase the muscle contraction-inhibitory effects of *Z. piperitum* extract and acetyl hexapeptide-8 by enhancing the absorption of them, however, but it was not the case. Although the result was negative, hyaluronic acid still has a possibility to enhance the effects of *Z. piperitum* extract and acetyl hexapeptide-8 in humans. Since *C. elegans* has relatively simple and premature skin structures compared to that of human^15^, hyaluronic acid treatment might have no effect. But in the case of humans, promoting skin absorption can enhance muscle contraction-inhibitory effects. It would be interesting to examine the synergy between *Z. piperitum* extract, acetyl hexapeptide-8, and hyaluronic acid in humans.

In this study, we investigated non-invasive cosmetic ingredients that can ameliorate symptoms of skin aging such as wrinkles. We identified *Z. piperitum* extract as a wrinkle-reducing reagent and determined the mechanism-of-action of *Z. piperitum* extract. Finally, we showed that *Z. piperitum* extract treatment decreased lateral canthal rhytides in humans. Considering the fact that the effect of BoNT/A injection is irreversible and lasts at least several months, treatment of *Z. piperitum* extract and/or acetyl hexapeptide-8 seems to be much safer than BoNT/A because it is fully reversible. Thus, we propose the combination of *Z. piperitum* fruit extract and acetyl hexapeptide-8 as a safe and non-invasive cosmetic application to improve expressional wrinkles.

## Materials and methods

### Strains, cell lines, and reagents

For cell culture experiments, C2C12 mouse skeletal myoblasts (ATCC CRL-1772), PC12 a pheochromocytoma of the rat adrenal medulla cells (ATCC CRL-1721), and NG108-15 mouse neuroblastoma x rat glioma cells (ATCC HB-12317) were purchased from the American Type Culture Collection (ATCC, Manassas, VA, USA).

The following media compositions were used for the subculture of corresponding cells:

- C2C12: DMEM with 10% fetal bovine serum, 1% penicillin–streptomycin
- NG108-15: DMEM (w/o sodium pyruvate) with 10% fetal bovine serum, 0.1 mM hypoxanthine, 400 nM aminopterin, 0.016 mM thymidine, 1.5 g/L sodium bicarbonate and 1% penicillin–streptomycin
- PC12: RPMI-1640 media with 10% heat-inactivated horse serum and 5% fetal bovine serum and 1% penicillin–streptomycin

All the reagents for preparing media were purchased from Gibco, USA.

The *Caenorhabditis elegans* (*C. elegans*) strain N2 wild-type, provided by Caenorhabditis Genetics Center, which is funded by the National Institutes of Health—Office of Research Infrastructure Programs (P40 OD010440), was examined in this study.

Other reagents were purchased as the following information; *Z. piperitum* fruit extract (OBMlab, Korea), BoNT/A (Innotox, Hugel, Korea), BoNT/A LC (Bioprogen, Korea), TD1-LC (BPMed Cosmetic, Korea), Acetyl hexapeptide-8 (Beadteach, Korea), Acetyl hexapeptide-8-FITC (Peptron, Korea), ultra-low molecular weight (0.5–10.1 kDa) hyaluronic acid (trade name: Oligo-HA, SK Bioland, Korea).

### Co-cultured cell-based muscle contraction assay

We performed muscle contraction assays as previously described^16^ with modifications. C2C12 myoblast cells and PC12 pheochromocytoma cells were used for the muscle contraction assay. Briefly, C2C12 cells were differentiated for 6 days in DMEM (Gibco, USA) containing 10% heat-inactivated horse serum (Gibco, USA) and 1% penicillin–streptomycin (Gibco, USA). Then the differentiated C2C12 cells were co-cultured with PC12 cells for 4 days in DMEM containing 10% heat-inactivated fetal bovine serum (Gibco, USA) and 1% penicillin–streptomycin. For the muscle contraction assay, co-cultured cells were washed gently with Phosphate-Buffered Saline (PBS, Gibco, USA), and treated with serum- and antibiotics-free DMEM containing specific concentrations of chemicals. The cells then were subjected to 20 V, 20 ms, 1 Hz of electric pulse stimulations for 30 min using a C-Pace pulse generator (C-Pace EP, IonOptix, USA). Muscle contraction rates were counted for 30 s by observing the contraction of muscle cells under a Leica DM IRM microscope (Leica, Germany), and converted to the number of contractions per minute.

### Muscle contraction assay in C. elegans

Muscle contraction assay was designed based on the ideas of swimming rate assay or motility assay described previously with modifications^17,18^. Briefly, synchronized young adult worms were harvested with M9 buffer and washed mildly. Worms were treated with specific concentrations of chemicals that were solubilized in 1 mL of M9 buffer for 1 h at 20 °C. After treatment, worms were washed twice with M9 buffer and transferred into 24-well plates containing 1 mL of fresh M9 buffer. After 1 min of equilibration, the movement of worms was observed by using Invitrogen EVOS FL Auto 2 (Thermo Fisher, USA). The body bends of individual worms were considered as one muscle contraction and the number of muscle contractions was counted for 30 s. The number of muscle contraction per minute were then calculated.

### Calcium influx assay

Calcium influx assay was performed by using the FLIPR Calcium Assay Kit (Molecular Devices, USA) as described previously^19^. NG108-15 cells were seeded on the poly-L-lysine-coated 96-well plate. After 3 days, cells were treated with *Z. piperitum* extract diluted in the serum-free DMEM for an hour. After *Z. piperitum* extract treatment, cells were treated with loading dye for 2 h. Intracellular calcium ion concentrations were examined upon 70 mM of KCl stimulation at 485/525 nm by using FLEXstation (Molecular Devices, USA).

### Neurotransmitter release

To examine neurotransmitter release, we applied the Neurotransmitter Transporter Uptake Assay Kit (Molecular Devices, USA) with modifications. After 3 days of co-cultivation of C2C12 and NG108-15 cells, cells were treated with dye solution for 30 min for cells to take up the neurotransmitter-mimic fluorescent dye. Next, cells were treated with *Z. piperitum* extract diluted with HBSS (with 20 mM HEPES, Gibco, USA) for 30 min. Neurotransmitter release was induced with 70 mM KCl treatment. The decrease of intracellular fluorescent levels was monitored by using FLEXstation (Molecular Devices, USA) at the 485/525 nm.

### Analysis of the skin absorption of FITC-tagged acetyl hexapeptide-8

Analysis of the skin absorption of FITC-tagged acetyl hexapeptide-8 was performed as described previously with some modifications^11,20^. Briefly, a reservoir of Franz diffusion cells was filled with PBS and porcine skin (Micropig, back skin, 2.5 × 2.5 cm^2^, 1 mm thickness, MediKinetics, Korea) was placed on the Franz diffusion cells. One hundred microliters of the aqueous solution containing FITC-tagged acetyl hexapeptide-8 were applied topically and then incubated at 37 °C with 50% relative humidity for 16 h. After incubation, to remove residual samples on the porcine skin, the surface of the porcine skin was wiped-off 3 times by using PBS-socked cotton swabs. To examine the amount of samples absorbed in the stratum corneum layer, the porcine skin was stripped-off 3 times with D-Squame tape (Eurofins Dermatest Pty Ltd, Australia). Each tape was soaked with 1 ml PBS, and incubated for 2–3 h at 50 °C to extract the samples absorbed in the stratum corneum. To examine the amount of samples absorbed in the epidermis and the dermis layers, tape-stripped porcine skin was placed in the plastic bag and then soaked in hot water (65 °C) for 30 s. Epidermis and dermis were separated by pushing the surface of the skin by using a flat iron spatula. Each tissue was homogenized by using a Precellys 24 homogenizer (Bertin Technologies, Montigny, France) and centrifuged (Centrifuge 5427R, Eppendorf, Hamburg, Germany) at 18,000 g for 10 min. Each supernatant was used for examining the amount of permeated FITC by using a fluorescence spectrometer (Varioskan Lux, Thermo Fisher Scientific, Waltham, MA, USA).

To determine the effect of hyaluronic acid on acetyl hexapeptide-8 absorption, porcine skin fixed with Franz diffusion cell was prepared as described above. Twenty microliters of the aqueous solution containing FITC-tagged acetyl hexapeptide-8 and ultra-low molecular weight hyaluronic acid were applied topically and then incubated at 37 °C with 50% relative humidity for 16 h. The formaldehyde-fixed paraffin sections were used for fluorescent imaging. FITC-tagged acetyl hexapeptide-8 was imaged by using Invitrogen EVOS FL Auto 2 (Thermo Fisher, USA). The fluorescent intensity in the stratum corneum layer or epidermis was quantified by using ImageJ (http://imagej.nih.gov/ij/).

### Clinical study

We recruited 24 healthy women aged over 38 years old. Of 24 healthy female volunteers who were initially included in the trial 23 completed the study and were included in the final analysis. The study was randomized, double-blinded, and placebo-controlled. The participants were divided into a placebo group (n = 8), *Z. piperitum* extract-treated group (n = 7, 60 ppm), and *Z. piperitum* extract (60 ppm) with acetyl hexapeptide-8 (50 ppm)-treated group (n = 8). Lateral canthal rhytides were evaluated after daily application of corresponding formulations for 0, 4, 8, 12 weeks by using a 3D skin imaging system PRIMOS High Resolution (Canfield Scientific GmbH, Germany). Images adjusted to the same position by 3D matching function and were analyzed by using the PRIMOS Lite software. The measurement variable Ra (an arithmetic average value of all heights and depths to the reference plane, µm unit) was used for evaluating wrinkles as previously described^21^. Percent wrinkle change was calculated based on the initial Ra value (Ra at the 0^th^ week).

### Ethics statement

The clinical study was conducted by the principles and guidelines expressed in the Declaration of Helsinki. The experimental design was approved by the institutional review board of LG Household and Health Care, Ltd. (LGHH-20200327-AA-01). The institutional review board is operated independently including external evaluation members following the Korean Bioethics and Safety Act and certified by the Ministry of Health and Welfare of Korea (Certification No.1–20,170,421,107-AB-N-01). All participants provided informed written consent prior to the study enrollment.

## Supplementary information

Supplementary figure S1

Supplementary figure S2

Supplementary figure S3

Supplementary figures

Supplementary files

Supplementary video 1

Supplementary video 2

Supplementary video 3

Supplementary video 4

## Data Availability

All data generated or analyzed during this study are included in the Supplementary Dataset. The original pictures or data generated during the clinical study are available from the corresponding authors on reasonable request.
