# Supplementary figures and images for: Topical application of Zanthoxylum piperitum extract improves lateral canthal rhytides by inhibiting muscle contractions
Source: Sci Rep. 2020 Dec 9;10:21514. doi: 10.1038/s41598-020-78610-w (PMC7726138; doi:10.1038/s41598-020-78610-w)

**Figure S1**

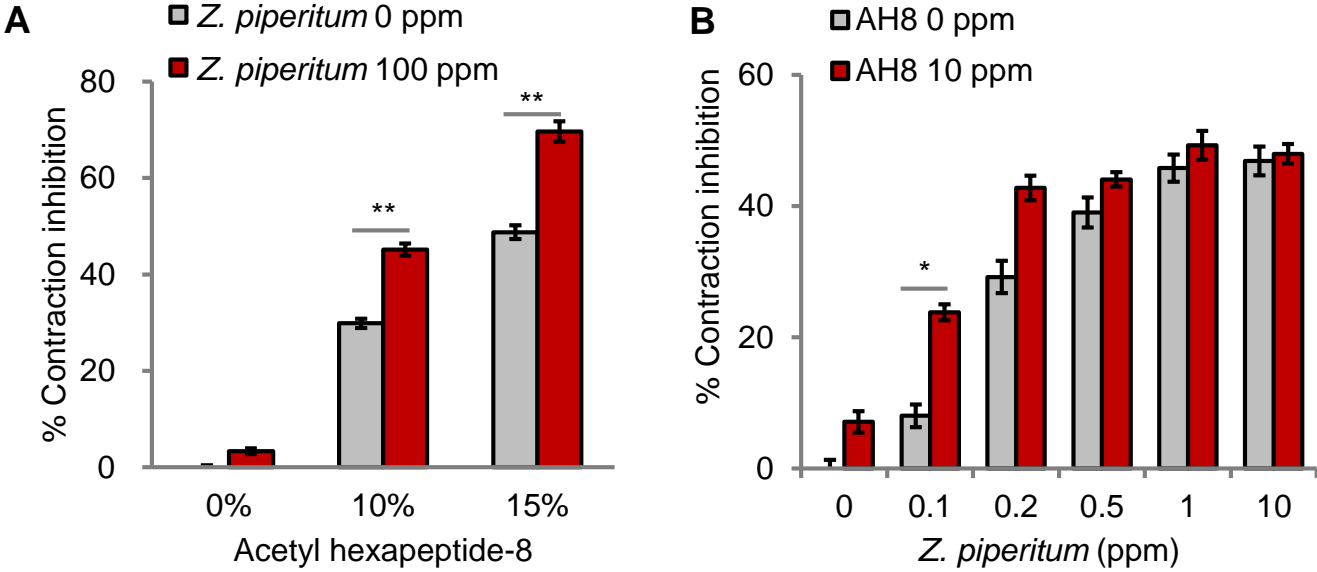

Supplement: Supplementary file 1 — Supplementary figure S1 [file 41598_2020_78610_MOESM1_ESM.pdf]

Figure S3

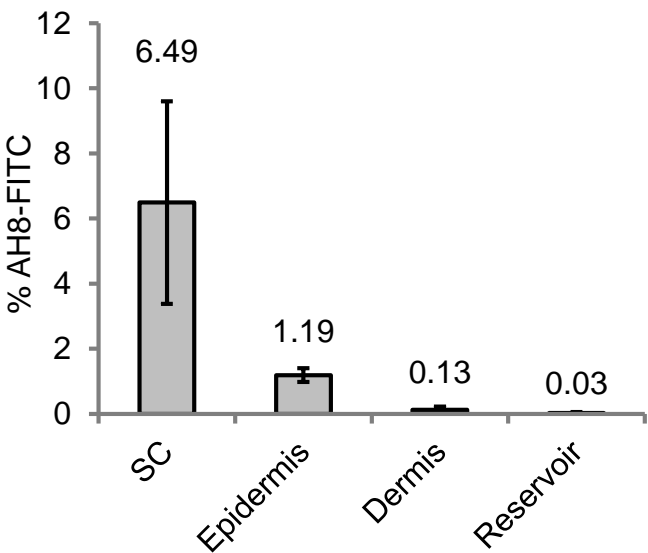

Supplement: Supplementary file 3 — Supplementary figure S3 [file 41598_2020_78610_MOESM3_ESM.pdf]
