# Supplementary material for: Topical application of Zanthoxylum piperitum extract improves lateral canthal rhytides by inhibiting muscle contractions
Source: Sci Rep. 2020 Dec 9;10:21514. doi: 10.1038/s41598-020-78610-w (PMC7726138; doi:10.1038/s41598-020-78610-w)
Supplement: Supplementary file 2 — Supplementary figure S2 [file 41598_2020_78610_MOESM2_ESM.pdf]

Figure S2

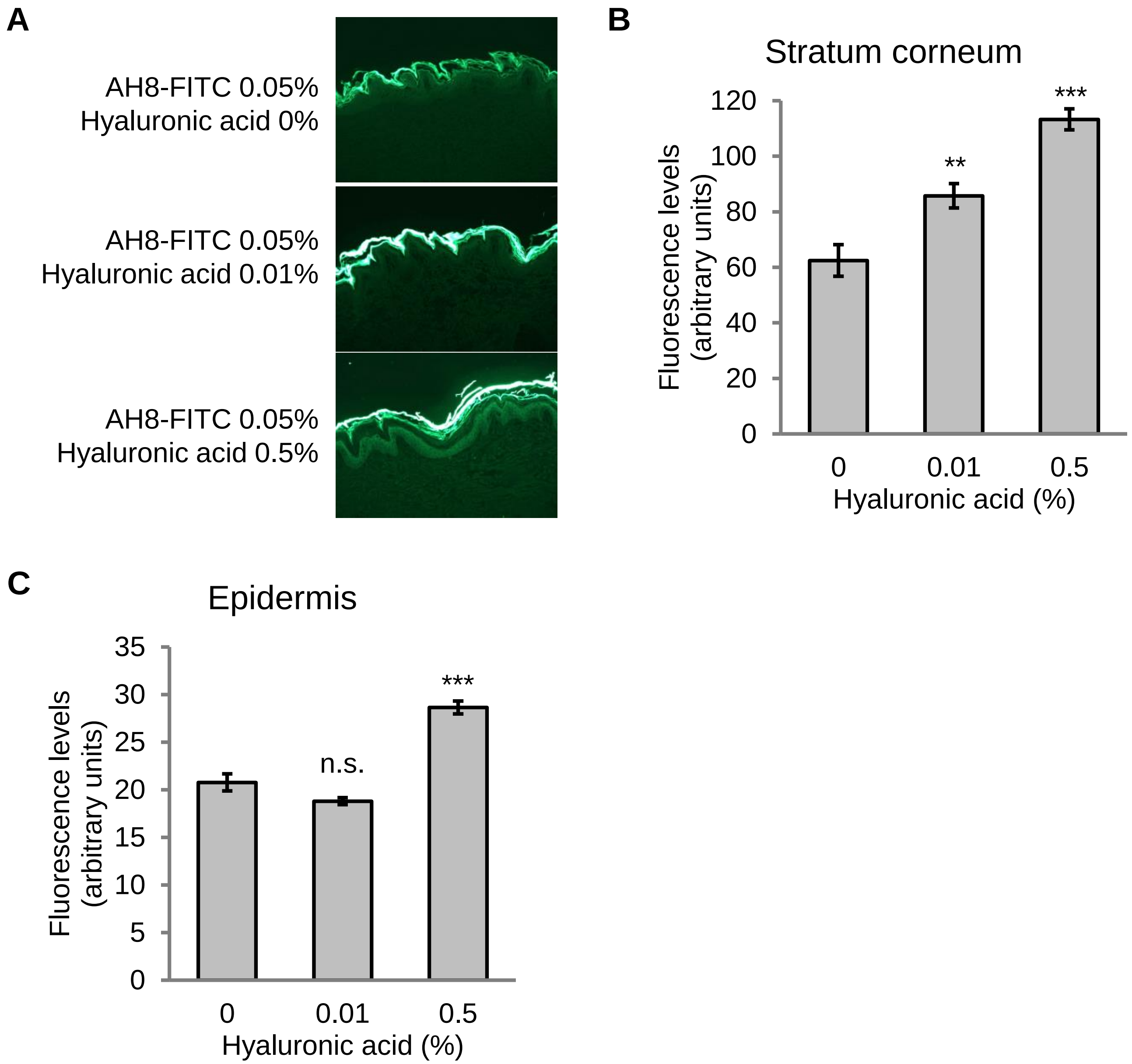
A
AH8-FITC 0.05%
Hyaluronic acid 0%
AH8-FITC 0.05%
Hyaluronic acid 0.01%
AH8-FITC 0.05%
Hyaluronic acid 0.5%
B
Stratum corneum
120
100
80
60
40
20
0
Fluorescence levels
(arbitrary units)
**
***
0
0.01
0.5
Hyaluronic acid (%)
C
Epidermis
35
30
25
20
15
10
5
0
Fluorescence levels
(arbitrary units)
n.s.
***
0
0.01
0.5
Hyaluronic acid (%)
