# Supplementary material for: Topical application of Zanthoxylum piperitum extract improves lateral canthal rhytides by inhibiting muscle contractions
Source: Sci Rep. 2020 Dec 9;10:21514. doi: 10.1038/s41598-020-78610-w (PMC7726138; doi:10.1038/s41598-020-78610-w)
Supplement: Supplementary file 5 — Supplementary files [file 41598_2020_78610_MOESM5_ESM.docx]

**Supplementary Information**

**Topical application of *Zanthoxylum piperitum* extract improves lateral canthal rhytides by inhibiting muscle contractions**

Wooseon Hwang^1, *^, Daehyun Kim^1^, Oh Sun Kwon^1^, Yun-Sun Kim^1^, Byungjun Ahn^1^, and Nae-Gyu Kang^1, *^

^1^R&D Center, LG Household and Healthcare, E10 building, LG Science Park, 70 Magokjungang-10-ro, Seoul, 07911, South Korea

*Co-corresponding authors. E-mail: wooseon@gmail.com, ngkang@lghnh.com

**Supplementary Figure Legends**

**Figure S1. Synergistic muscle contraction inhibition by *Z. piperitum* extract and acetyl hexapeptide-8**

(**A**) High concentrations (10% and 15%) of acetyl hexapeptide-8 treatment had synergistic effects with 100 ppm of *Z. piperitum* extracts on muscle contraction inhibitions in *C. elegans.* (**B**) Confirmation of the synergistic effects of *Z. piperitum* extract and acetyl hexapeptide-8 in cell-culture system. All the experiments were performed at least twice. Error bars represent standard error of mean (S.E.M., *p < 0.05, **p < 0.01, two‐tailed Student's t‐test).

**Figure S2. Hyaluronic acid increases the absorption of acetyl hexapeptide-8**

(**A**) Ultra-low molecular weight hyaluronic acid increased the skin absorption rate of acetyl hexapeptide-8. We used FITC-tagged acetyl hexapeptide-8 (AH8-FITC). Consistent with the previous report^11^, 0.5% of hyaluronic acid increased the absorption of acetyl hexapeptide-8 at the epidermis layer. We found that 0.01% of hyaluronic acid increased the skin absorption of acetyl hexapeptide-8, especially at the stratum corneum layer, but not at the epidermis layer. (**B** and **C**) Quantification of figure (**A**). All the experiments were repeated at least twice. Error bars represent standard error of mean (S.E.M., n.s.: not significant, **p < 0.01, ***p < 0.001, two‐tailed Student's t‐test).

**Figure S3. Quantification of the basal absorption ratio of topically applied acetyl hexapeptide-8**

All the experiments were repeated three times. Error bars represent standard error of mean (S.E.M.).

**Supplementary Video 1. An example of muscle contraction of differentiated muscle cells (control)**

**Supplementary Video 2. An example of muscle contraction of differentiated muscle cells treated with acetyl hexapeptide-8**

**Supplementary Video 3. An example of a muscle contraction of *C. elegans* (control)**

**Supplementary Video 4. An example of muscle contraction of *C. elegans* treated with acetyl hexapeptide-8**
